# Non-medical and non-invasive interventions for erectile dysfunction in men with type 2 diabetes mellitus: A scoping review

**DOI:** 10.1016/j.heliyon.2023.e15778

**Published:** 2023-04-28

**Authors:** Setho Hadisuyatmana, James H. Boyd, Ferry Efendi, Gulzar Malik, Michael Bauer, Sonia Reisenhofer

**Affiliations:** a School of Psychology & Public Health, La Trobe University, Kingsbury Drive, Bundoora, Victoria, 3086, Australia; b Faculty of Nursing, Universitas Airlangga Indonesia, Kampus C Jln Mulyorejo, 60115, Surabaya, East Java, Indonesia; c School of Psychology and Public Health, La Trobe University of Australia, Kingsbury Drive, Bundoora, Victoria, 3086, Australia; d Honorary Fellow at La Trobe University of Australia, Kampus C Jln Mulyorejo, 60115, Surabaya, East Java, Indonesia; e School of Nursing & Midwifery, La Trobe University of Australia, Kingsbury Drive, Bundoora, Victoria, 3086, Australia; f School of Engineering, La Trobe University of Australia, Kingsbury Drive, Bundoora, Victoria, 3086, Australia; g Bairnsdale Regional Health Service, Victoria, Australia, 122 Day St, Bairnsdale, VIC, 3875, Australia

**Keywords:** Type-2 diabetes mellitus, Erectile dysfunction, Patient education, Lifestyle, Diet modification, Physical exercise, Therapy, Vacuum erectile device, Low-intensity extracorporeal shock wave therapy

## Abstract

**Background:**

Erectile dysfunction (ED) often affects men with type 2 diabetes mellitus (T2DM) due to microvascular damage. However, medical interventions are not always appropriate.

**Aim:**

This scoping review aimed to answer the following question: What evidence is available about the effects of non-medical and non-invasive healthcare interventions to improve ED in men with T2DM?

**Method:**

Potential studies were collected from the Cumulative Index to Nursing and Allied Health Literature via EBSCO, Embase via Ovid, MEDLINE via Ovid, Web of Science, PubMed, ProQuest, and PsycINFO via Ovid.

**Findings:**

From 2,611 identified titles, 17 studies, including 11 interventional and 6 observational studies, were included. Four main alternatives to medical interventions were identified from the included studies. Amongst these, four studies recommended patient education on lifestyle modification, twelve studies encouraged dietary changes and physical activities, two studies emphasized the use of vacuum erectile device, and three studies suggested the application of low-intensity extracorporeal shockwave therapy by healthcare professionals.

**Discussion:**

Dietary modification and physical activities were promoted as effective interventions to help maintaining the erectile function in men with T2DM. Several methods of patient education were identified as the approach to facilitate lifestyle modification in men with T2DM-associated ED. The positive outcomes of this review support early ED screening to help preventing T2DM complications such as ED in men. Further, T2DM management is a shared responsibility between the men and healthcare professionals. Despite the success of Vacuum Erectile Device and Low-intensity Extracorporeal Shockwave Therapy in regaining erectile function, further research is needed in this area based on the recommendations of the American Urological Association. Moreover, the health and quality of life of men with T2DM must be improved.

## Highlights

• Not all medical interventions are appropriate for men with T2DM-associated erectile dysfunction.
• Invasive treatments are not always feasible or desirable options for erectile dysfunction.
• Educational programs can facilitate lifestyle modification.
• Dietary change and physical activity are important for regaining erectile function.

## 1 Introduction

Erectile dysfunction (ED), defined as the failure to attain and/or maintain penile erection, is closely associated with type 2 diabetes mellitus (T2DM) [1]. Men with T2DM are at three times higher risk of ED than those without diabetes [2,3]. The persistent hyperglycemia present in T2DM eventually disrupts penile vascularization and damages nerve endings [4]. This leads to fiber and autonomic neuropathies and small artery impairment, all of which contribute to ED [5]. Persistent insulin resistance, on the other hand, is responsible for reduced hypothalamic response to endogenous insulin, thereby causing hypogonadotropic hypogonadism [6,7] and testosterone deficiency in men with T2DM [2]. In addition, the ED can be triggered by anti-hypertensives and anti-depressants that are commonly prescribed to patients with T2DM [8].

Previous studies have shown that ED can develop within the first 5 years following T2DM diagnosis [9]. However, most men were not aware of the issue until the damage had started to affect their personal lives [10]. Thus, the close association between T2DM-associated ED (T2DMED), psychological stress and depression can significantly affect men's quality of life [8,[11], [12], [13]].

In men, T2DMED can cause sexual dissatisfaction and distress [14], unsatisfactory relationships, and marital tension [15,16]. An earlier study found that T2DMED created frustration and anger in men which were directed to their wives [15]. Nevertheless, T2DMED screening is inconsistent, and the provision of support to the men remains a challenging issue [[17], [18], [19]].

The use of phosphodiesterase-5 inhibitors (PDE5I) is widely recommended as safe and effective in regaining erectile function [8,20]. However, some men do not respond to PDE5I. Further, invasive treatments may not be desirable nor feasible in some cases. Therefore, this scoping review was conducted to identify non-medical and non-invasive alternatives for maintaining erectile function in men with T2DM.

## 2 Material and methods

### 2.1 Design

This scoping review was undertaken to summarize evidence-based alternatives for the non-medical management of T2DMED [[21], [22], [23]]. With regard to its nature as a secondary study, this scoping review did not require an ethical approval. The review was conducted in five consecutive stages, as described by Arksey and O'Malley (2005) [21]. These included: (1) identifying the research question, (2) determining relevant studies, (3) selecting relevant studies, (4) charting data, and (5) collating, summarizing, and reporting study results [21]. The conduct of this review was documented and reported based on the Preferred Reporting Items of Systematic Reviews and Meta-Analyses for Scoping Reviews (PRISMA ScR) checklist (Supplementary File 3) [24].

### 2.2 Establishing the research question

This review aimed to answer the following question: What evidence is available regarding non-medical and non-invasive interventions for managing erectile dysfunction in men with T2DM?

### 2.3 Identifying relevant studies

A set of key terms was developed to maintain research consistency. The terms were cross-checked and expanded using the Medical Subject Headings available in https://meshb.nlm.nih.gov/search for article inclusion (Table 1).

**Table 1:** Key words used for searching relevant studies in this review.

**•** (‘erectile dysfunction*' OR ‘sexual activit*' OR ‘sexual dysfunction*' OR ‘sexual malfunction*' OR ‘sexual disorder*' OR ‘hypoactive sexual desire*' OR ‘premature ejaculation’ OR ‘delayed ejaculation’ OR ‘physical discomfort’) AND (‘Type-2 Diabetes' OR ‘Diabetes mellitus type 2' OR ‘Diabetes Mellitus' OR DM or Hyperglycaemia OR ‘adult onset diabetes' OR ‘metabolic disorder*')

**Abbreviations:** DM = Diabetes Mellitus.

In February 2022, authors evaluated studies published in the following databases: Cumulative Index to Nursing and Allied Health Literature via EBSCO, Embase via Ovid, MEDLINE via Ovid, Web of Science, PubMed, ProQuest, and PsycINFO via Ovid. These databases were selected for their collection of published articles in health and related sciences. The identification of potential additions was made by hand-searching the reference lists of the included studies. However, this review did not consider gray literature for inclusion. The search strings used to identify potential articles for inclusion and the search history is presented in supplementary file 1 and 2.

### 2.4 Study selection

Eligible studies, including peer-reviewed studies on men diagnosed with T2DMED that were written in English and articles published from January 2002 to February 2022, were assessed for inclusion (Table 2). All processes in this study selection stage (title, abstract, and full-text review) were independently conducted by two reviewers using an online platform Covidence® (www.covidence.org). The work of this stage was carefully recorded using the 2020's PRISMA ScR flow diagram [24].

**Table 2:** Criteria for inclusion of published articles.

Participants
Men who were diagnosed with T2DM and experience ED.
Intervention
Non-pharmacological (i.e., non-medical, noninvasive) interventions used to address T2DMED
Study design
Peer-reviewed, quantitative studies, including:
Trials (randomized or non-randomized controlled trials, comparative studies, single-arm treatment group studies, cohorts); and
Cross-sectional studies, retrospective studies
Outcomes
Delayed ED, steady erectile function (or IIEF scores), improved erectile function (or IIEF scores)

**Abbreviations:** ED = Erectile Dysfunction; IIEF = International Index of Erectile Function; T2DM = Type-2 Diabetes Mellitus; T2DMED = Type-2 Diabetes Mellitus associated Erectile Dysfunction.

### 2.5 Charting the data

The data extracted from the included studies were charted in a table as informed by the Joanna Briggs Institute's Manual for the Conduct of Scoping Reviews [25]. This table provides article details, including name of author(s), publication year, journal volume and issue number, study setting(s), participants/sample details, method(s), intervention(s), and key findings (Table 4).

**Table 3:** Included articles for extraction.

Author(s), year	Journal, vol. (issue)	Study setting(s)	sample (y.o)	Method(s) & data analysis	Key interventions & findings/results (*)
El-Sakka, Sayed and Tayeb (2009).^1^	Urology, 74 (3)	Saudi Arabia	159 (40–76)	Single-Arm Prospective Study (no control) (Unpaired *t*-test & one-way ANOVA)	**Key intervention(s):** Diabetes control instituted using lifestyle modification (diet control and physical exercise) in addition to hypoglycaemic agents and/or insulin therapy. Follow-up of scheduled visits to the diabetologist clinic every 4 weeks to adjust the *anti*-DM treatment according to patients’ response and tolerance. At 3- and 6-month follow-up visits, patients were assessed for the same parameters of baseline visit. **Key finding(s):** At baseline visit, 22% of patients had normal T level and good metabolic control; this percentage increased to 28.9% and 29.6% at 3- and 6-month follow-up visits (P = 0.001, OR: 95% CI = 3.1 (2.2–4.1). Improvement in Mean IIEF score from baseline visit, 3-month visit, to 6-month visit (12.4 + 28; 15.3 + 3.3; 17.8 + 4.5, respectively), with P = 0.001 (baseline vs six-month follow up).
Esposito et al. (2006).^**2**^	Journal of Sexual Medicine, 7 (5)	Italy	611 (35–70)	Cross-sectional (chi-square test, simple linear regression)	**Key intervention(s):** Patients were invited to follow their usual oral treatment and/or insulin. The study investigated the association of men's adherence to Mediterranean diet and their erectile function. **Key finding(s):** The men with the highest adherence score to the Mediterranean diet had the lowest global prevalence of ED when compared with the middle and lowest tertile, respectively (52.6% v 59.1% v 61.2, P = 0.01).
Khatana et al. (2008).^**3**^	International Journal of Impotence Research, 20 (5)	The USA	41 (62.4 ± 9.7)	Retrospective analysis of an earlier Randomized Control Trial (paired Student's t-tests or two-tail Fisher's exact tests)	**Key intervention(s):** Participants in the treatment arm attended weekly group sessions of MEDIC for one month, where clinical pharmacists provided education to intensify behavioural modification (i.e., encourage diet, exercise and disease self-management, tobacco cessation and other comorbid risk reduction) and medication titration followed algorithms to achieve tobacco cessation, glycaemic, blood pressure, and lipid control. **Key finding(s):** Participants who had met goals for both blood pressure and HbA1c experienced the greatest improvement in IIEF-5 (2.2 ± 1.6) followed by participants who met only one of the goals (BP or HbA1c) (−0.05 ± 0.8), and then by those meeting none of the goals (−1.5 ± 1.2). Changes in systolic and diastolic BP were still significant predictors of change in IIEF-5 after adjusting for age (P = 0.01 and P = 0.01, respectively) and change in PDE5I use (P = 0.01 and P = 0.01, respectively).
Khoo et al. (2011).^4^	Journal of Sexual Medicine, 8 (10)	Australia	31 ((58.1 ± 11.4) – (62.3 ± 5.9))	Comparative study (independent samples *t*-test)	**Key intervention(s):** All subjects had glycated haemoglobin on diet control or oral hypoglycemic medication. Subjects were randomly assigned to one of two different diets: modified Low-calorie diet (LCD) or a high-protein low-fat diet (HP diet). The LCD group consumed two sachets daily (one at breakfast and lunch or dinner) of a liquid meal replacement (Kicstart, Pharmacy Health Solutions, Sydney, Australia), providing a maximum of 450 kcal of energy, 0.8 g/kg ideal body weight of protein, and the recommended daily allowances of minerals, vitamins, and omega-3 and omega-6 essential fatty acids, plus one other small meal, for a total of ∼900 kcal/day. The HP diet was prescribed to reduce daily energy intake by ∼600 kcal, including 300 g of lean meat/poultry/fish, and three servings/day of cereals/bread and low-fat dairy foods and two fruit and five vegetable serves per day. **Key finding(s):** At 8 weeks, there was a significant increase in the mean IIEF-5 score in both LCD and HP groups, with no difference between the groups. Of the 17 men (54.8%) with severe ED (IIEF-5 score <8) at baseline, seven demonstrated improvements in IIEF-5 scores. At 52 weeks, further improvements in IIEF-5 score were seen in both groups (Table 2), main effect of time F (2, 28.1) = 41.55, P < 0.001. Normal IIEF-5 score (>21) was achieved in four men (25.0%) at 12 months, compared to none at the start of the study and one in the LCD group at 8 weeks
Kirilmaz et al. (2015).^**5**^	The Aging Male, 18 (4)	Turkey	83 (26–75)	Randomized Control Trial (*t*-test, paired *t*-test, Kruskal-Wallis test)	**Key intervention(s):** Patients in control group received lifestyle modifications and intense glycemic control. Lifestyle modification consisted of a moderate physical activity of 30–45min for 3–5 days per week (walking or exercises that increase the movement in joints), and a Mediterranean diet that includes a low amount of saturated fats and red meat, and is rich in grains, dry legumes, green vegetables, and fruits with olive oil being the oil source. Likewise, patients in treatment group received the similar lifestyle modification consisted of a moderate physical activity of 30–45min for 3–5 days per week and a Mediterranean diet. In addition, they also received oral PDE5i (Viagra, Pfizer, Turkey) two or three times per week during 3 months of trial. **Key finding(s):** When the patients were evaluated regarding the improvement in their erectile function, 23 of 41 patients (44.2%) in Group 1 and 29 of 42 patients (55.8%) in Group 2 were found to have increased IIEF-5 scores (p = 0.321). The evaluation of changes in the IIEF-5 scores revealed that the mean increase was 2.5 in Group 1, and 5 in Group 2, and the change in IIEF-5 scores were more significant in Group 2 (p = 0.012).
Maiorino et al. (2016).^6^	Journal of Diabetes and Its Complications, 30 (8)	Italy	106 (53.1)	Randomized Control Trial (one-sample *t*-test and Wilcoxon signed rank test)	**Key intervention(s):** Newly diagnosed patients who had never treated with antidiabetic drugs were randomly assigned to received Mediterranean diet or a low-fat diet, to restrict energy intake to 1800 kcal/day. The Mediterranean diet had the goal of no more than 50% of calories from carbohydrates and no less than 30% calories from fat, with the main source of added fat 30–50 g of olive oil. The low-fat diet had the goal of no more than 30% of calories from fat and no more than 10% of calories from saturated fat. **Key finding(s):** Participants in the low-fat group experienced a greater reduction of IIEF (mean score 19.6, mean difference: −2.23, P < 0.001) than those in the Mediterranean diet group (mean score 20.8, mean difference: −1.22, P < 0.001) the absolute between-group difference (Mediterranean diet vs low-fat diet) in IIEF was 1.16, P = 0.024). At the end of 8.1 years of trial, male diabetic patients with the highest scores of adherence to Mediterranean diet (scores of 6–9) had higher IIEF values than diabetic patients who scored by points on the scale: 21.9 (SD, 2.3), 20.7 (2.4), and 19.7 (2.2) for the high, middle, and low adherence scores, respectively (P for trend = 0.010).
Pajovic et al. (2017).^7^	The Aging Male, 20 (1)	Montenegro	50 (35–67)	Comparative study (student's t-test)	**Key intervention(s):** Patients were advised to use VED besides their regular and standard therapy for diabetes mellitus. The patients were recommended to use the device as prescribed and at least four times per month. Erectile function was evaluated prior the treatment, as well as six months after using VED. **Key finding(s):** 85% participants reported their achievement of regaining erectile function to allow normal sexual intercourse, while 15% others did not have satisfying penis rigidity.
Rosen et al. (2009).^8^	Journal of Sexual Medicine, 6 (5)	The USA	390 (45–75)	Cross-sectional (Student's unpaired t-tests and ANOVA, Bivariate and multivariate analyses, forward logistic regression models, Correlational analyses, Hosmer and Lemeshow goodness-of-fit test)	**Key intervention(s):** To assess the correlation of exercise fitness with the tendency of ED. **Key finding(s):** Exercise fitness levels at baseline, including both unadjusted and age-adjusted fitness levels (maximum METS; age-adjusted z- scores), were protective of ED (Max METS OR = 0.76; CI: 0.65–0.85; z- adjusted fitness OR = 0.61; CI: 0.47–0.78). Men with improved levels of fitness were about 40% less likely to have ED compared with those in the lowest group
Shendy et al. (2021).^**9**^	Andrologia, 53 (4)	Egypt	42 (41–55)	Randomized Control Trial (independent sample *t*-test, paired *t*-test, Pearson product-moment)	**Key intervention(s):** Patients in the treatment group were treated with Li-ESWT plus Pelvic Floor Exercise (PFE). Patients in the control group were treated with PFE (Kegel exercises three times daily for 6 weeks) and sham therapy by a shock wave. **Key finding(s):** The IIEF-EF increased significantly in the SW group (Baseline 12.75 + 3.21 to EOT 17.50 + 2.72, p < 0.001), but not in the control group (Baseline 12.75 + 2.61 to EOT 13.40 + 2.85, p = 0.194). Comparing the post-treatment IIEF between the two groups, it was higher in the treatment group, and this was statistically significant (p < 0.001). In the Li-ESWT group, 15 (71%) patients achieved erection sufficient for penetrative intercourse versus 2 (9.5%) patients in the control group (p < 0.001).
Sun et al. (2014).^10^	International Journal of Urology, 21 (12)	China	60 (age detail not available)	Randomized Control Trial (student's t-test)	**Key intervention(s):** Patients in control group were instructed on the proper use of a VED by personal tutoring and watching an instructional video (Timm Medical Technologies; Eden Prairie, MN, USA), and given a device for use without the constrictor ring; a battery-operated pump is then applied to create an artificial erection. Once the desired state of erection is achieved, the erect state is maintained for 1–2 min, released and then pumped again; this is practiced for at least 30 min every day, and the patients use a constriction ring during coitus, but this should not be left on for longer than 30 min. Patients in treatment group underwent VED combined with sildenafil therapy. They received sildenafil 100 mg daily 1 h before sexual activity or 2 h after a meal and were given the same course of VED as the control group. **Key finding(s):** During the 1-month and 3-month visits, the scores were significantly higher for the sildenafil plus VED group than for the VED group (14.86 ± 2.17 vs 12.41 ± 2.63; P < 0.0001) and (17.53 ± 2.95 vs 14.29 ± 2.81; P < 0.0001). Men in the treatment group had better successful penetration (43.7% vs 30% and 73.3% vs 46.6%) successful intercourse (43.3% vs 26.6% and 70% vs 46.6%) at the 1-month and 3-months visits compared with the control group. Combination therapy significantly improved erectile function compared with the VED-only group.
Wing et al. (2010).^11^	Journal of Sexual Medicine, 7 (1)	The USA	372 (45–74)	Randomized Control Trial (ANOVA, ANCOVA)	**Key intervention(s):** Participants were randomly assigned within clinic to the Intensive Lifestyle Intervention (ILI) or to a control group that received diabetes support and education (DSE). The ILI focused on changing diet and physical activity with a study goal of inducing a loss of initial weight during year 1. Participants attended weekly meetings during months 1–6 and were seen 3 times/month during months 7–12. Intervention included a combination of group and individual sessions modelled on group behavioural weight loss programs and on approaches used successfully in the Diabetes Prevention Program (DPP). Calorie restriction was the primary approach to weight loss; participants were given calorie goals of 1200–1500 if < 250 lb and 1500–1800 if > 250 lb, with a maximum of 30% of calories from fat. The physical activity recommendation was to gradually increase activity to 175 min/week, using moderate intensity activities such as brisk walking. The DSE group attended an initial diabetes education course and was invited during year 1 to three sessions that provided basic education about diet and physical activity, as well as opportunities for social support. Participants in both groups continued to receive their medical care from their own physicians. **Key finding(s):** After adjusting for baseline differences in EF score, there was only a trend for men in the ILI group to have greater increases in EF than men in DSE (+1.3 ± 4.7 vs. + 0.03 ± 5.7; p = 0.06). In DSE, 20% reported worsening in their ED category (e.g., moving from moderate erectile dysfunction at baseline to severe dysfunction at 1-year), 57% stayed in the same category, and 23% reported improvements in category. The distribution in ILI differed significantly (p = 0.006), with only 8% reporting worsening, 70% staying in the same category, and 22% reporting improvements.
Yaman et al. (2006).^12^	Journal of Sexual Medicine, 3 (2)	Turkey	17 (31–62)	Single-Arm Prospective Study (no control) (paired *t*-test, Wilcoxon signed ranks test, and Mann–Whitney *U* test)	**Key intervention(s):** All patients were provided with a specific diabetes education program and were seen in the outpatient unit every 2–4 weeks for 6 months of duration. Dietary intervention was intensified in all patients. Patients were also advised to do physical exercise properly. Glycemic optimization was achieved with insulin either as monotherapy or combined with oral hypoglycemic agents in type 2 patients in appropriate fashion. **Key finding(s):** No statistically significant difference for IIEF scores and NPTR parameters (including number of erections, duration of erectile episode, duration of episode tip rigidity >60%, RAU tip, TAU tip, RAU base, TAU base) was found either before or after treatment (6 months after regulation) and in patient subgroups according to FPG (<140 mg/dL vs. >180 mg/dL), HbA1c (≤8.1% vs. >8.1%), type of DM (type 1 vs. type 2), and duration of DM (<10 years vs. ≥10 years) (P > 0.05).
Geyik^13^	Andrologia	Turkey	18 of 41 (51.61 ± 11.80)	Restrospective evaluation (no control) (independent *t*-test)	**Key intervention(s):** All patients included in this study had been subjected to a total of two courses of Li-ESWT apart six months. One course consisted of five implementations about 7 ± 2 days apart. In each implementation, 1800 shockwaves (SW) (0.09 mJ/mm2) were applied to the distal penile shafts and 1800 SW to the perineal corpus cavernosum. The treatment was administered in an outpatient setting without anaesthesia, wherein the application areas were the same, and each implementation lasted approximately 20 min. **Key finding(s):** For patients with Diabetes, mean IIEF-5 scores were 13.94 ± 3.87 at baseline, 20.72 ± 4.31 after the first course (MD: −6.600; 95% CI, −8.965: −4.235; t = −6.312; p < 0.0001) and 23.94 ± 4.78 after the second course (MD: −2.700; 95% CI, −3.379: −2.021; t = −9.000; p < 0.0001). The power of positive correlation between the baseline and after first course scores was 0.76 and also the power of positive correlation was 0.98 between the first and the second courses. Both p-values were significant with <0.05. The evaluation of successful treatment rates was 56% in patients with DM
Giagulli et al. (2020).^14^	Andrology, 8 (3)	Italy	71 (50.3 ± 3.3)	Retrospective evaluation (no control)	**Key intervention(s):** A hypocaloric Mediterranean diet (1500 ± 200 kcal; 50% carbohydrate, 20% protein, and 30% fat) and a brisk walk three times per week for a cumulative time of 150 min per week are prescribed to each patient, when needed. In patients with uncontrolled T2DM on lifestyle intervention, we use metformin as the first-line medication. **Key finding(s):** Thirty-five (49%) out of 71 patients achieved improvements in IIEF-5. Patients with a body weight loss <5% had a lower prevalence of improvement in IIEF-5 when compared to those achieving a body weight loss of 5%–10% or >10% (30% vs 65% and 61%, respectively; P = 0.02).
Minami et al. (2018).^15^	Journal of Diabetes Investigation, 9 (1)	Japan	460 (60.8–11.6)	Cross-sectional (logistic regression, Mul- tiple regression logistic analyses)	**Key intervention(s):** This study investigated the association between levels of physical activities in men with T2DM, with regard to the severity of ED. **Key finding(s):** After adjustment for confounding factors, walking habit was independently inversely associated with ED, (adjusted ORs 0.62, 95% CI: 0.39–0.979 and 0.63, 95% CI: 0.41–0.98, respectively). Exercise habit was independently inversely associated with severe ED, but not moderate-to-severe ED (adjusted OR 0.52, 95% CI: 0.32–0.82). Walking habit in the study indicates 3 METs/h36, and the amount of weekly physical activities were ≥21 METS/hour/week. Walking habit was independently inversely associated with moderate-to-severe ED and severe ED. Thus, might have a protective effect on erectile function.
Verze et al. (2020).^16^	Asian Journal of Andrology, 2020 (22)	Italy	156 (56.0 ± 9.6), (58.2 ± 3.2)	Retrospective comparative (Student's t-test, Mann–Whitney *U* test)	**Key intervention(s):** Patients in the treatment group received LiESWT during the first 3 weeks of treatment with tadalafil. Shockwaves were delivered to the distal, mid, and proximal penile shaft, as well as to the left and right crura. The duration of each LiESWT session was about 20 min. Energy density was set at 0.09 mJ mm−2 and frequency at 120 min−1. The number of shockwaves delivered during each session varied from 1500 to 2400. Treatment protocol consisted of two treatment sessions per week for 3 weeks, 3 days apart within the week. The control group received tadalafil 5 mg once daily bedtime alone for 12 weeks **Key finding(s):** A statistically significant improvement of mean IIEF-5 scores with respect to baseline was evident in all groups at 4-week follow-up. In details, the IIEF-5 scores (mean ± SD) improved by 3.9 ± 1.9 and by 2.9 ± 1.5 in treatment and control group, respectively. At 24-week follow-up, the IIEF-5 (mean ± SD) variations with respect to baseline in treatment and control group were +3.8 ± 2.4 and +1.8 ± 1.7, respectively (P < 0.001 in both groups). These results demonstrate that the combined approach provides advantages in terms of both magnitude of mean IIEF-5 score improvement and durability of results if compared to tadalafil 5 mg once daily alone.
Khoo et al. (2010).^17^	International Journal of Obesity, 2010 (34)	Australia	51 (19 person with dieabetes 58.1 ± 11.4)		**Key intervention(s):** Subjects in the intervention group were asked to consume 2–3 sachets daily (one at breakfast and lunch and/or dinner) of Kicstart, providing a maximum of 450 kcal of energy, 0.8 g per kg of ideal body weight of high-quality protein, as well as the recommended daily allowances of minerals, vitamins, trace elements, omega-3 and omega −6 essential fatty acids. One small meal at either lunch or dinner was permitted, consisting of ‘noncarbohydrate’ vegetables and a small piece of meat, fish or chicken, to achieve a total energy intake of approximately 850–900 kcal per day. Each subject was given the same meal plan with details of specific allowable foods and portions and was able to contact the dietician in between follow-up visits to answer queries about the diet. **Key finding(s):** After 8 weeks, the diabetic subjects achieved significant weight loss (9.5 ± 4.8 kg, P < 0.01), reduced waist circumference (12.1 ± 4.8 cm, P < 0.01), and an improvement in insulin sensitivity (percentage change in QUICKI) (9.0 ± 9.1%, P < 0.01) in response to the low-calorie diets. A significant increasement in the mean IIEF-5 score were seen in diabetic subjects (2.1 ± 3.0, P < 0.01) in response to weight loss induced by the LCD. In diabetic men, the absolute increase in the IIEF-5 score was similar to that of the nondiabetic intervention subjects but represented a proportionally greater improvement from baseline. The average SDI scores also increased significantly in the and diabetic subjects (10.4 ± 9.4, P < 0.01). Significant reductions in mean IPSS scores were seen in diabetic subjects (−2.1 ± 2.3, P < 0.01)

Abbreviations: CI = Confidence Interval; DM = Diabetes Mellitus; DSE = Diabetes Support and Education; DPP = Diabetes Prevention Program; ED = Erectile Dysfunction; EF = Erectile Function; EOT = End of Trial; FPG = Fasting Plasma Glucose; HbA1c = Haemoglobin A1c level; HP = High-Protein Low-Fat Diet; IIEF(-5) = International Index of Erectile Function (version 5); ILI = Intensive Lifestyle Intervention; IPSS = International Prostate Symptom Score; LCD = Low-Calorie Diet; Li-ESWT = Low-intensity Extracorporeal Shock Wave Therapy; METS = Metabolic Equivalent Score; MEDIC = Multidisciplinary Education in Diabetes and Intervention for Cardiac risk reduction; NPTR = Nocturnal Penile Tumescence And Rigidity Test/Score; OR = Odds ratio; P = Probability value; PDE5I = Phosphodiesterase-5 inhibitors; PFE = Pelvic Floor Exercise; SD = Standard Deviation; SDI = Sexual Desire Inventory; SW = Shockwave(s); VED = Vacuum Erectile Device; RAU = Rigidity Activated Units; TAU = Tumescence Activated Units.

**Table 4:** Non-pharmacological interventions and outcomes identified from the included studies.

Authors, year	Non-pharmacological approach						Medication	Duration of treatment (month)				IIEF Score		
	DM	PE	BM	Edu	VED	LiESWT		<3	3 to 6	>6 to 12	≥12	Decrease	Steady	Increase
El-Sakka, Sayed & Tayeb [60]	*	*					*		*					*
Giugliano et al. [61]	*						*	*						*
Khatana et al. [62]				*			*	*						*
Khoo et al. [63]	*						*			*				*
Kirilmaz et al. [64]	*	*					*		*					*
Maiorino et al. [65]	*	*									*		*	
Pajovic et al. [66]		*			*		*		*					*
Rosen et al. [67]		*					*						*	
Shendy et al. [68]		*				*			*					*
Sun et al. [69]					*		*		*					*
Wing et al. [70]				*			*			*		*	*	*
Yaman et al. [71]				*			*		*				*	
Geyik [72]						*			*					*
Giagulli et al. [73]	*	*					*			*				*
Minami et al. [74]		*											*	
Verze et al. [75]						*	*		*					*
Khoo et al. [76]	*	*						*						*

**Abbreviations:**DM = Diet modification; PE = Physical exercise; BM = Behavioral Modification, including disease self-management, tobacco cessation and other comorbid risk reduction; Edu = Patient education sessions; VED = Vacuum Erectile Device; LiESWT = Low-intensity Extracorporeal Shockwave Therapy; PFE = Pelvic Floor Exercise/Kegel; IIEF = International Index of Erectile Function.

### 2.6 Collating, summarizing, and reporting data

Extracted data from included studies were coded and analyzed inductively into themes. Based on the accepted approach of scoping reviews, the identified evidence were then summarized without being critically appraised [21].

## 3 Results

### 3.1 Included studies

**Fig. 1:**
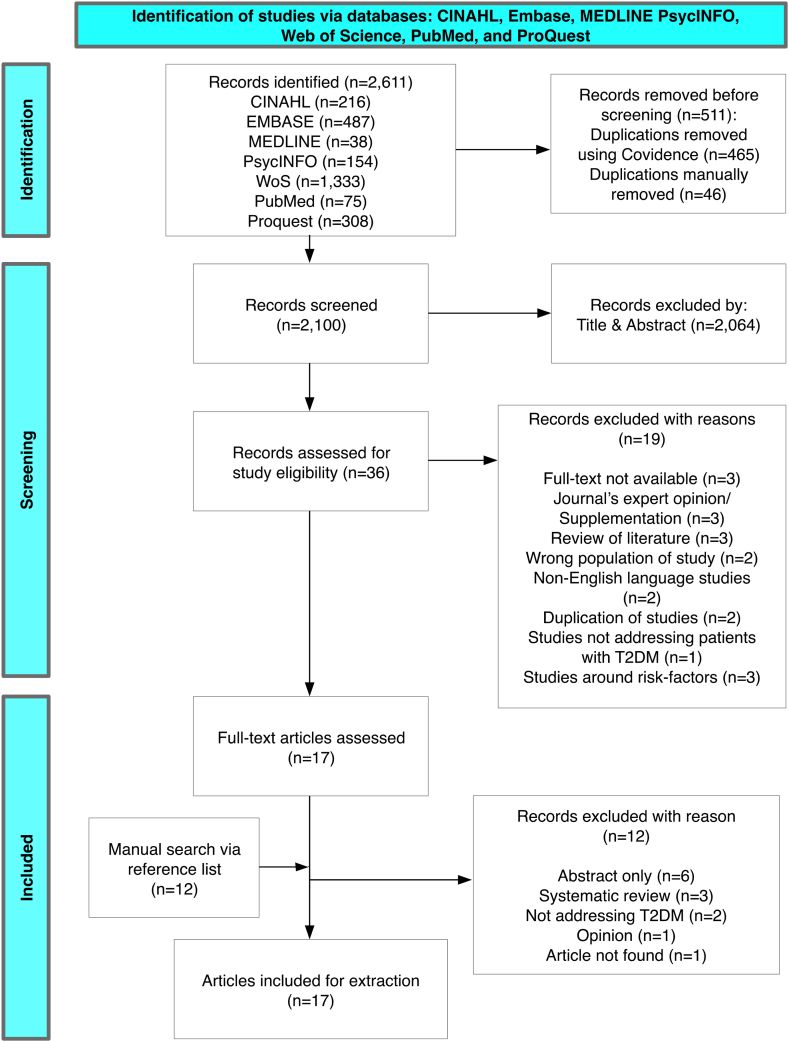
Flow diagram of the search in the scoping review works based on 2020's PRISMA ScR.

At its final stage, this review included 17 studies eligible for data extraction, with details in the following description and the 2020 PRISMA ScR diagram to illustrate the conducted processes (Fig. 1).

### 3.2 Title review

The search initially yielded 2,611 titles, which were then exported to Covidence® for duplication removal. This step removed 511 duplicated titles, thereby leaving 2,100 titles for manual abstract review.

### 3.3 Abstract review

Two reviewers individually screened the abstracts of the remaining titles in Covidence®. The third and fourth reviewer were invited to resolve conflicts for final decisions. At this stage, 2,064 records were excluded due to irrelevant topics identified from the abstracts.

### 3.4 Full-text review

At this stage, 36 records were retained for full-text review. Nineteen articles were then excluded based on the inclusion criteria, full-text availability, and duplication of studies (Fig. 1). The reference lists of the included articles were hand-searched. However, none were eligible for additional inclusion. At the end of this stage, 17 primary studies were included for data extraction (Table 3).

### 3.5 Descriptive findings of the included studies

This scoping review extracted evidence that explored different non-pharmaceutical and non-invasive approaches for regaining or maintaining erectile function in men with T2DM. With regards to the diverse evidence, this review maps the included studies based on (a) year of publication, (b) country of origin, and (c) study methods.

### 3.6 Publication year

**Fig. 2:**
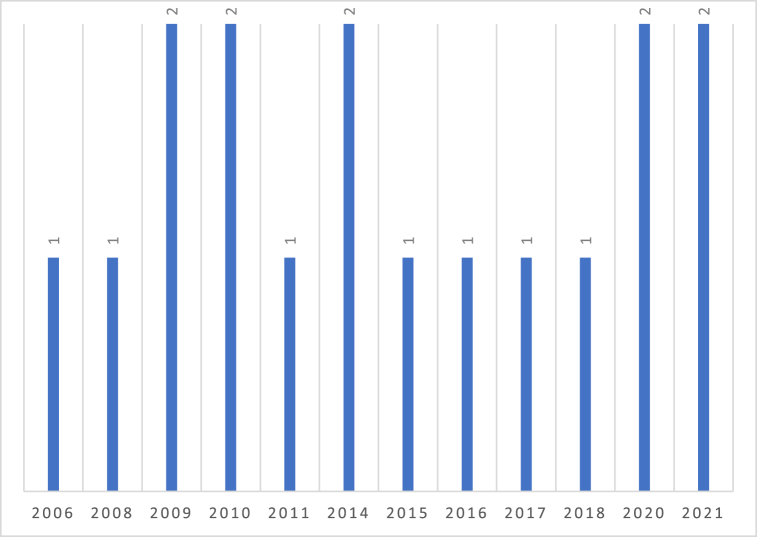
Articles by year of publication.

This review did not identify any relevant study published between 2001 and 2006. Only studies published between 2006 and 2021 were included for data extraction (Fig. 2). Of all the included studies, 58.8% (n = 10) were published in the last decade [[26], [27], [28], [29], [30], [31], [32], [33], [34], [35], [36]], with the rest (n = 7) published before 2011 [[37], [38], [39], [40], [41], [42], [43]].

### 3.7 Origin of studies

**Fig. 3:**
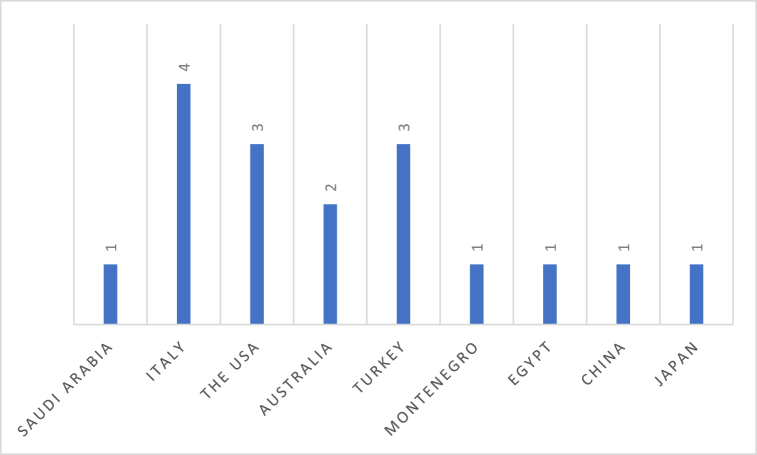
Included studies by setting/country of origin.

Regarding the country of origin, all studies originated from middle- and high-income countries. Up to 47.05% (n = 8) of the included studies were originated from European countries [26,27,29,30,32,35,36,38,43] and a small proportion of the studies (n = 2) were conducted in East Asian countries, including Japan [31] and the People's Republic of China [34] (Fig. 3). This suggests that no studies have been conducted in developing countries.

### 3.8 Study methods

**Fig. 4:**
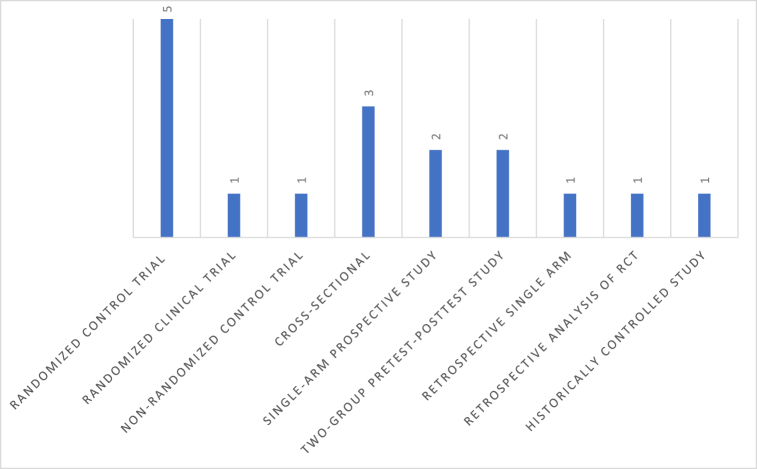
Included studies by design/method.

The included studies have a wide variety of methods. In total, 11 were interventional studies, including: five randomized controlled trials (RCTs) [29,30,33,34,42], one non-randomized controlled trial [40], one randomized clinical trial [28], and four pre- and post-intervention studies [26,32,37,43]. The remaining studies were cross-sectionals [31,38,41] and retrospective studies [27,36,39] (Fig. 4). With regards to the length of the conducted trials (Table 4), the shortest study was 1 month [38] and the longest observation was conducted for 8.1 years [30]. Both of these studies advocated a combination of Mediterranean diet and physical exercises to men who were newly diagnosed with T2DM, either as adjunct to oral anti-diabetic therapies [38] or as a single approach for those who had never been prescribed with any medication [30].

### 3.9 Alternatives to non-medical and non-invasive interventions

This review summarizes the alternatives of non-medical and non-invasive treatments identified in the included studies. The studies were then grouped into themes based on the approaches suggested for maintaining and regaining ED in men who were diagnosed with T2DM. The themes included dietary modifications and physical exercises, with additions from a small number of studies that promoted the use of a device or medical assistance to help men achieve erection for sexual penetration, including the use of assistive vacuum erectile device (VED) and low-intensity extracorporeal shockwave therapy (Li-ESWT) (Table 4). More importantly, all studies highlighted the importance of patient education in facilitating the treatments [39,40,42,43]. However, most of the included studies proposed the interventions in conjunction with hypoglycemic agents [27,28,32,[37], [38], [39], [40],^42^,^43^] and/or PDE5I [29,34,36,41]. These interventions were grouped as follows:

1. Patient education
2. Lifestyle modification
3. Assistive device as a treatment modality
4. Energy based therapy

### 3.10 Patient education

Different methods of patient education, which can support and facilitate dietary changes and prescribed physical exercises in men, were promoted in four studies [39,40,42,43]. Two studies originating from the USA showed the involvement of multidisciplinary health professionals in patient education [39,42]. This method was used to improve the fundamental awareness of participants on T2DM and associated comorbidities (including ED) and the adoption of healthier lifestyle.

A retrospective analysis of RCTs investigated the significance of a serial multi-professional education referred as Multidisciplinary Education in Diabetes and Intervention for Cardiac Risk Reduction (MEDIC) in empowering affected men to follow the recommended lifestyle modification [39]. The intervention, as described by Martin et al. (2007), was a package of group training sessions that consisted of four weekly sessions (for up to 20 patients) [44]. Each training session comprised of a 90-min education session around T2DM self-management delivered by healthcare professionals (HCP) and a 60-min workshop to assist men in setting goals to achieve behavioral and medication modifications. Based on the MEDIC study, each HCP's roles and participations are explained as follows:

1. Clinical pharmacists explain the medication, risk factors of cardiovascular events, and importance of regular risk monitoring.
2. Nurse educators provide instructions and trainings on using monitoring devices, such as glucometer, pedometer, sphygmomanometer, and other necessary skills for diabetes self-care.
3. Nutritionists are involved in introducing food groups, label reading, and using essential skills to control weight, blood glucose levels, and blood pressure through controlled diet.
4. Social workers as consultants discuss the challenges experienced by men and the strategies that must be used to overcome psychological barriers.
5. Physical therapists are involved to explain safe exercises, skills and tools for heart rate monitoring, and to suggest exercise equipment [44].

Another RCT investigated the effect of intensive lifestyle interventions (ILI) for T2DM that were introduced from a larger and earlier study conducted by The Look AHEAD Research Group [45]. Originally, the ILI was designed as a long-term lifestyle modification tool that includes the following.

#### 3.10.1 Phase 1: at 0–6 months

1. In total, 24 sessions of individual and group treatment focusing on behavioural management, calorie intake recording, dietary modification, and increased physical activities.
2. The group sessions are conducted in the first three weeks of the month.
3. Individual appointments in the fourth week of the month.

#### 3.10.2 Phase 2: at 7–12 months

1. Two group meetings and one individual session of meeting per month.
2. The meetings are focused to individualize personal diet and maintain the minimum duration of moderate-level physical activities (175 min per week).

#### 3.10.3 Phase 3: at 2–4 years

1. Two individual monthly updates with a lifestyle counsellor for support and monitoring

#### 3.10.4 Phase 4: at ≥ 5 years

1. Monthly individual meeting with a lifestyle counsellor
2. Reduced frequency of meeting to twice a year at minimum
3. Advisable biennial refreshment session

However, the included RCT was limited to evaluate outcomes after the first year of the ILI program (phase 1 and 2), particularly to identify its significance on weight loss and erectile function [42]. The dietary modification was designed to restrict calorie intake to 1500 kcal/day for participants weighing up to 113.4 kg and up to 1800 kcal/day for those weighing f more than 113.4 kg [42]. In addition, moderate-intensity exercises, such as brisk walking, was gradually increased up to 175 min per week.

Another study conducted in Turkey investigated the significance of a six-month specific diabetes education program on the erectile function of men diagnosed with T2DM [43]. The education sessions were delivered either fortnightly or monthly to institute dietary modification and adaptation of a proper physical exercise, as complementary to the prescribed anti-hyperglycemic agents (such as metformin, sulfonylureas, and glitazone) and/or other therapies.

Another educational approach was used in a non-RCT study in Australia to assist men with T2DMED in substituting their regular diet by consuming liquid meals [40]. A dietician was involved in establishing a detailed meal plan to identify allowable food and portion, as well as to assist the implementation and monitor the progress at fortnightly basis. The two-month interventions of the trial was principally identified as follows.

1. Two to three sachets of Kicstart (Pharmacy Health Solutions Pty Ltd., Sydney, Australia) to be consumed daily as substitutes to breakfast and lunch or dinner.
2. One small meal comprising “non carbohydrate” vegetables and a small piece of fish or chicken at dinner or lunch can be consumed to achieve a total energy intake of approximately 900 kcal/day.

### 3.11 Lifestyle modification

Most studies that promoted lifestyle modification maintained existing medication regimes (oral hypoglycemic agents or other therapies) [[27], [28], [29],^34^,[36], [37], [38], [39], [40], [41], [42], [43],^46^]. In total, 12 studies included dietary modification and physical exercise as additional therapies to the existing medical intervention to help men preserve or regain erectile function [[27], [28], [29],^34^,[36], [37], [38], [39], [40], [41], [42], [43],^46^]. These include four studies that introduced lifestyle modification in addition to PDE5I therapy to regaining erectile function [29,34,41]. Some of these studies concluded that the inclusion of dietary modification and physical exercise is effective in improving erectile function [28,31,38,41]. This suggests that diet control and physical activity can be used as complementary interventions to pharmacological approaches [27,37,40].

Another study promoted dietary modification and physical exercise as an effective single approach for men who were newly diagnosed with T2DM and had never been prescribed with antihyperglycemic agents [30]. The study emphasized that a Mediterranean diet is protective against the progression of T2DM and the associated ED (box 1). In addition, the study recommended 30-min of daily aerobic exercise for optimum outcomes [30].

Box 1 **Brief detail of Mediterranean diet as explained in Esposito et al. (2016) and Maiorino et al. (2016)**

Main goal.

1. Calorie intake restriction only up to 1800 kcal/day for men and1500 kcal/day for women.
2. Proportion:

Approximate daily intake of 50%–60% calories from carbohydrates, 15%–20% calories from protein, 30% calories from fat, 10% calories from saturated fat, and no more than 300 mg of cholesterol.

3. Recommended daily intake:

250–300 g of fruit, 125–150 g of vegetables, 25–50 g of nuts, 400 g of whole grains (such as legumes, rice, maize and wheat), and olive oil.

Alt-text: Box 1

### 3.12 Assistive device as a treatment modality

This review identified the use of assistive devices that can be prescribed by medical practitioners as second-line alternatives to regain erectile function. VED was identified in two studies as an assistive device that can be prescribed to the men [32,34]. However, different approaches were used in the studies. An RCT originating from China reported improvements in erectile function after 3 months of using the VED following individual tutorial session and an instructional video on how to safely use the device for artificial erection (box 2) [34]. The study reported greater improvement in erectile function in men who used the VED as an addition to PDE5I [34].

Box 2 **Basic principles on the use of VED, as described by Sun et al. (2014)**

1. Without the constrictor ring, use the battery-operated pump to create an artificial erection.
2. Once the desired state of erection is achieved, maintain the pump for 1–2 min and then release.
3. Repeat the use for at least 30 min per day or use the constrictor ring for coitus.
4. Only use the constrictor ring for 30 min to prevent injury.

Alt-text: Box 2

In another study, Pajovic et al. (2017) prescribed the use of VED to men with T2DM who had been unsuccessful with PDE5I [32]. The study involved men who were diagnosed with T2DM less than five years and cautiously excluded those who received anticoagulants or those with cardiovascular risks, penile anomaly, prostate disease, and endocrinological complications. The VED was prescribed as an addition to regular and standard T2DM medical therapies and/or lifestyle modification [32]. Further, the study suggested the minimum use of four times per month and the results to be evaluated after the sixth month.

### 3.13 Energy based therapy

Low-intensity extracorporeal shockwave therapy (Li-ESWT) was identified as a supportive treatment that should be administered by medical specialists [26,33,36]. In a study conducted in Italy, Verze et al. (2020) investigated the use of Li-ESWT as a combination therapy with a 5 mg daily administration of tadalafil in patients with T2DM and ED [36]. The study was designed to evaluate the significance of a six serialtreatments of Li-ESWT with 3-day intervals. The therapy was applied using Omnispec ED1000 (Medispec Ltd., Yehud, Israel) onto the distal, middle, and proximal areas of the penile shaft, and given on both sides of the crura (penile base); with the density of 0.09 mJ/mm^2^and the frequency of 120 shocks/minute for 20 min per session [36].

In the second study conducted in Egypt, a double-blind RCT was undertaken to evaluate the outcomes of Li-ESWT and pelvic floor exercise (PFE) in comparison with PFE in addition to sham therapy [33]. Both the sham and Li-ESWT were delivered twice per week for 3 weeks and were then repeated after a 3 week resting period [33]. Meanwhile, the PFE was prescribed three times per day for 6 weeks [33].

The third study was performed in Turkey to evaluate the outcomes of shockwave therapies in patients who had previously received two courses of Li-ESWT [26]. Each course comprised of five sessions with the average interval of 7 ± 2 day using Linear Renova (Initia Ltd., Petah Tikva), with 20 min of 0.09 mJ/mm^2^ intensity on the distal penile shafts. The three studies shared similar criteria, such as excluding men with glycated hemoglobin levels (HbA1C) > 7 mg/mL, hypogonadism, non-adjusted cardiac and antihypertensive medications, penile abnormalities, and history of pelvic surgeries.

### 3.14 Results and outcome measures

All studies used the International Index of Erectile Function (IIEF) as the tool to evaluate the outcomes of interventions, particularly erectile function. Four studies [30,32,41,42] used the original version of IIEF [47], and the remaining [[26], [27], [28], [29],^31^,^33^,^34^,[36], [37], [38], [39], [40],^43^] utilized the IIEF-5 [48]. Most studies offered the interventions alongside existing anti-diabetes agents (n = 9, 52.9%]) [27,28,32,[37], [38], [39], [40],^42^,^43^] or PDE5I (n = 4 [23.5%], i.e., Viagra [Pfizer], sildenafil, and tadalafil) [29,34,36,41]. Thirteen (76.4%) studies reported successful results, as indicated by increased IIEF scores [[26], [27], [28], [29],[32], [33], [34],[36], [37], [38], [39], [40],^42^]. Meanwhile, five studies reported steady IIEF scores, suggesting that the treatments have protective effects against ED [30,31,[41], [42], [43]].

Although, some studies reported negative outcomes, for example, Wing et al., reported the worsening ED in 8% of their study sample following lifestyle modifications [42], some argued that this is due to old age, overweight/obesity [42], and later stage T2DM [32,43].

## 4 Discussion

This scoping review summarizes evidence extracted from published studies that investigated complementary and alternative interventions to help men with T2DM maintain and/or regain erectile function. Adaptation to healthier lifestyle, including dietary modifications and physical exercises, was the most offered approach identified in the included studies. In addition, a small number of studies introduced VED and Li-ESWT as supportive treatments to regaining erectile function. The findings of this review further supported the need for health professionals to address T2DMED, with regards to the low rates of ED screening in health appointments [8,20,49].

Patient education was identified as the main approach used in promoting lifestyle modification (i.e., dietary change and physical activity) and other measures for improving T2DM management and erectile function [[27], [28], [29], [30],^37^,^39^,^40^,^42^,^43^]. Some of the included studies have shown that dietary modifications and physical activities (such as aerobic workouts, strengthening exercise, or a combination of the two) are safe and effective therapies for improving general health, diabetes management, and erectile function [27,29,30]. Most studies reported significant outcomes from lifestyle modification as additional therapy to PDE5I. However, regaining erectile function is only possible after correcting body weight (i.e., overweight and obesity) and maintaining the optimum blood sugar level [50]. In addition, the prescribed use of VED and Li-ESWT was reported as potential modalities to help the men in regaining erectile function [26,[32], [33], [34], [35], [36]].

Studies originated from middle- and high-income countries, such as Italy, the USA, Turkey, China, Egypt, and Australia, showed the magnitude of the issue and the growing awareness of T2DMED in those countries. However, a gap was identified from the absence of such studies in developing countries and the small number of included studies in the present review. Hence, more studies are recommended in future.

### 4.1 Patient education and lifestyle modification

The present review emphasizes the significance of patient education in adopting healthy lifestyle (dietary change and physical activity) for men with T2DM. The promoted lifestyle modifications identified in the studies is effective in preserving erectile function in men newly diagnosed with T2DM. Thus, such approaches should be adopted as early as possible following T2DM diagnosis [51,52], to lower the risk of ED and other related complications.

Patient education has always been recommended as the initial approach for improving T2DM screening uptake and management [53]. Improved knowledge will direct patient awareness on T2DM and increase treatment adherence [54]. The European Association of Urology recommends education as an essential approach that will prevent misleading information, which is the primary cause of mismanagement and further unnecessary burdens, including T2DMED [52,54]. As emphasized by Katana et al. (2008) and Wing et al. (2010), the multidisciplinary involvement of professionals (including medical physicians, dieticians, physical therapists, pharmacists, nurses, and social workers) is significant in patient education and instituting healthier lifestyle to men with T2DMED. Therefore, a collaborative approach should be considered as a way to improve the sexual and general well-being of men with T2DM [39,42]. This approach was recommended by the American Diabetes Association in the 2019 and 2020 Standards of Medical Care in Diabetes [55,56].

Most studies in this review reported improvements in erectile function following lifestyle modification in addition to pharmacological approaches. However, one study revealed that lifestyle modification can be effective as a single intervention to protect the men against ED. Particularly, in individuals who were newly diagnosed with T2DM and had never received anti-diabetic agents [30]. Healthy diet, balanced energy intake, weight loss, improved blood pressure, better glycemic control, and physical conditioning are some of the outcomes that lead to the improved testosterone level and overall erectile function [27,28,30,[37], [38], [39], [40], [41], [42], [43]]. Nonetheless, this review identified a small number of cases where the prescribed treatments were not proven to be effective [42,43]. These studies argued that the undesirable results are attributed to old age and the longer duration of poorly managed T2DM [42,43]. Hence, damage had already progressed and irreversibly impacted men who had lived with longer periods of T2DM [51,57]. All these suggest the need for the early diagnosis of T2DM, identification of T2DMED, and initiation of lifestyle modifications [35,58,59]. It also supports the need for interactions between the men and the HCPs to facilitate ED screening and discussion [8,20,49].

### 4.2 Courses of treatment modality

The prescribed use of VED and courses of Li-ESWT are identified as alternative modalities for preserving erectile function in men with T2DM [26,[32], [33], [34], [35]]. The VED was suggested as a safe and effective self-performed therapy before considering invasive procedures [34]. In the study, Pajovic et al. (2017) reported the 85% success rate of VED in helping the men to regain satisfying artificial erection, including in those who were not on pharmacological therapies [32]. The artificial erection was facilitated by the negative pressure created to improve temporary penile blood flow, similar to normal penile erection [34]. However, one should consider the possible issues associated with the device, which include penis base coldness, pain, petechial bleeding, and penile numbness [32,33]. For these reasons, the American Urological Association (AUA) recommended a moderate-level use of VED n (evidence level: grade C) [50]; and suggested medical advice and supervision [32,34].

Unlike the VED, the course of Li-ESWT should only be administered by medical specialists [26,33,36]. Li-ESWT is identified as a supportive therapy to either pelvic floor exercise (Kegel) or daily consumption of PDE5I [26,33,36]. Although the benefits of Li-ESWT remained questionable (evidence level: grade C) [50]. The methods (i.e., VED and Li-ESWT), however, do not improve the underlying pathology (i.e., diabetes mellitus), which is key in managing T2DMED [50,52]. Therefore, the course of either VED or Li-ESWT should be based on a careful consideration [50].

### 4.3 Strengths and limitations

The present review has limitations due to its nature as a secondary study and the quality of the work. First, the evidence identified in published studies cannot draw emerging topics or produce novel findings. Rather, a scoping review is used as a method to summarize or map available evidence on a particular topic [21,23]. Second, the limited analysis that focused on managing T2DMED was attributed to small numbers of identified studies that were then included in this review. Therefore, future studies on the topic are recommended to address this gap. For instance, exploration of the factors affecting early diagnosis of T2DMED or treatments offered in healthcare settings. Third, the exclusion of gray literature could have limited the potential reports or other papers that could have provided a valuable information on the topic. However, this review followed rigorous processes, based on a widely accepted framework [[21], [22], [23]].

## 5 Conclusions

This review summarizes non-medical and non-invasive alternatives for T2DMED. Further, the evidence was extracted from the included studies using a widely accepted framework. Lifestyle modifications including dietary change and physical exercise, either as a single approach or combined with medical therapy, were identified as havingt positive effects against ED in men who were newly diagnosed with T2DM. Patient education is widely offered to facilitate lifestyle modification in men with T2DM.

The positive outcomes of this review support the initiation of ED screening and discussion to lower the risks of future complications of T2DM in men. The application of multidisciplinary approaches in educating patients about managing T2DM requires a collective and shared responsibility among HCPs. In addition, the use of VED and Li-ESWT is offered as an additional alternative to allow satisfactory erection, despite recommendation for further investigation of these treatments. Thus, the findings of this review should be used in future studies that aim to improve the health and quality of life of men with T2DM.

## Authors contribution statement

Setho Hadisuyatmana, Sonia Reisenhofer: Conceived and designed the experiments; Performed the experiments; Contributed reagents, materials, analysis tools or data; Analyzed and interpreted the data; Wrote the paper. Ferry Efendi, Michael Bauer: Conceived and designed the experiments; Analyzed and interpreted the data. James Boyd, Gulzar Malik: Analyzed and interpreted the data; Wrote the paper.

## Data availability

Data will be made available on request.

## Declaration of competing interest

The authors declare that they have no known competing financial interests or personal relationships that could have appeared to influence the work reported in this paper.
