# Supplementary material for: Non-medical and non-invasive interventions for erectile dysfunction in men with type 2 diabetes mellitus: A scoping review
Source: Heliyon. 2023 Apr 28;9(5):e15778. doi: 10.1016/j.heliyon.2023.e15778 (PMC10176068; doi:10.1016/j.heliyon.2023.e15778)
Supplement: Multimedia component 1 [file mmc1.docx]

Supplementary 1: Search Strings

### Database:

### Cumulative Index to Nursing and Allied Health Literature (CINAHL) via EBSCO, Embase via Ovid, MEDLINE via Ovid, Web of Science, PubMed, ProQuest and PsycINFO via Ovid.

| # | **Filters** | **Filters: Full-text, English, year 2002-current, NOT: review**, **book, report, single case, documents, opinion.** |
| --- | --- | --- |
| 1 | Concept 1: erectile dysfunction | ('sexual activit*' OR 'sexual dysfunction*' OR 'sexual malfunction*' OR 'sexual disorder*' OR 'erectile dysfunction*' OR ‘hypoactive sexual desire*' OR 'premature ejaculation' OR 'delayed ejaculation' OR 'physical discomfort').mp. [mp=title, abstract, original title, name of substance word, subject heading word, floating sub-heading word, keyword heading word, protocol supplementary concept word, rare disease supplementary concept word, unique identifier, synonyms] |
| 2 | Concept 2: diabetes mellitus | ('Type-2 Diabetes' OR 'Diabetes mellitus type 2' OR 'Diabetes Mellitus' OR DM or Hyperglycaemia OR 'adult onset diabetes' OR 'metabolic disorder*').mp. [mp=title, abstract, original title, name of substance word, subject heading word, floating sub-heading word, keyword heading word, protocol supplementary concept word, rare disease supplementary concept word, unique identifier, synonyms] |
| 3 | Concept 3: | exp Sexual Dysfunctions, Psychological/ OR exp Erectile Dysfunction/ OR exp Sexual Dysfunction, Physiological/ |
| 4 | Concept 4: | exp Diabetes Mellitus, Type 2/ |
| 5 | Search #5 | #1 AND #3 |
| 6 | Search #6 | #2 OR #4 |
| 7 | Search #7 | #4 AND #5 |
