# Supplementary material for: Non-medical and non-invasive interventions for erectile dysfunction in men with type 2 diabetes mellitus: A scoping review
Source: Heliyon. 2023 Apr 28;9(5):e15778. doi: 10.1016/j.heliyon.2023.e15778 (PMC10176068; doi:10.1016/j.heliyon.2023.e15778)
Supplement: Multimedia component 2 [file mmc2.docx]

Supplementary 2: Search Results & History

### Database #1: Cumulative Index to Nursing and Allied Health Literature (CINAHL) via EBSCO

| # | **Filters** | **Filters: Full-text, English, year 2002-current, NOT: review**, **book, report, single case, documents, opinion.** | **n** |
| --- | --- | --- | --- |
| 1 | Concept 1: erectile dysfunction | 'sexual activit*' OR 'sexual dysfunction*' OR 'sexual malfunction*' OR 'sexual disorder*' OR 'erectile dysfunction*' OR ‘hypoactive sexual desire*' OR 'premature ejaculation' OR 'delayed ejaculation' OR 'physical discomfort' | 4,597 |
| 2 | Concept 2: diabetes mellitus | 'Type-2 Diabetes' OR 'Diabetes mellitus type 2' OR 'Diabetes Mellitus' OR DM or Hyperglycaemia OR 'adult onset diabetes' OR 'metabolic disorder*’ | 52,353 |
| 3 | Limiters | English Language; Published Date: 20020101-; Human; Sex: Male; Language: English | |
| 4 | Expanders | Apply equivalent subjects | |
| 5 | Search modes | Boolean/Phrase | |
| 6 | Search | #1 AND #2 | **216** |

### Database #2: Embase via Ovid

| # | **Filters** | **Filters: Full-text, English, year 2002-current, NOT: review**, **book, report, single case, documents, opinion.** | **n** |
| --- | --- | --- | --- |
| 1 | Concept 1: erectile dysfunction | ('sexual activit*' or 'sexual dysfunction*' or 'sexual malfunction*' or 'sexual disorder*' or 'erectile dysfunction*' or 'hypoactive sexual desire*' or 'premature ejaculation' or 'delayed ejaculation' or 'physical discomfort').mp. [mp=title, abstract, heading word, drug trade name, original title, device manufacturer, drug manufacturer, device trade name, keyword heading word, floating subheading word, candidate term word] | 92,751 |
| 2 | Concept 2: diabetes mellitus | ('Type-2 Diabetes' or 'Diabetes mellitus type 2' or 'Diabetes Mellitus' or DM or Hyperglycaemia or 'adult onset diabetes' or 'metabolic disorder*').mp. [mp=title, abstract, heading word, drug trade name, original title, device manufacturer, drug manufacturer, device trade name, keyword heading word, floating subheading word, candidate term word] | 1,441,642 |
| 3 | Concept 3: | limit 1 to (human and male and english language and "therapy (best balance of sensitivity and specificity)" and english and yr="2002 -Current" and article and journal) | 3,261 |
| 4 | Concept 4: | (human and male and english language and "therapy (best balance of sensitivity and specificity)" and english and yr="2002 -Current" and article and journal) | 45,186 |
| 5 | Search #5 | #3 AND #4 | **487** |

### Database #3: MEDLINE via Ovid

| # | **Filters** | **Filters: Full-text, English, year 2002-current, NOT: review**, **book, report, single case, documents, opinion.** | **n** |
| --- | --- | --- | --- |
| 1 | Concept 1: erectile dysfunction | ('sexual activit*' or 'sexual dysfunction*' or 'sexual malfunction*' or 'sexual disorder*' or 'erectile dysfunction*' or 'hypoactive sexual desire*' or 'premature ejaculation' or 'delayed ejaculation' or 'physical discomfort').mp. [mp=title, abstract, original title, name of substance word, subject heading word, floating sub-heading word, keyword heading word, organism supplementary concept word, protocol supplementary concept word, rare disease supplementary concept word, unique identifier, synonyms]  exp Erectile Dysfunction/ | 20,123 |
| 2 | Concept 2: diabetes mellitus | ('Type-2 Diabetes' or 'Diabetes mellitus type 2' or 'Diabetes Mellitus' or DM or Hyperglycaemia or 'adult onset diabetes' or 'metabolic disorder*').mp. [mp=title, abstract, original title, name of substance word, subject heading word, floating sub-heading word, keyword heading word, organism supplementary concept word, protocol supplementary concept word, rare disease supplementary concept word, unique identifier, synonyms]  Diabetes Mellitus, Type 2/ | 152,338 |
| 3 | Concept 3: | limit 1 to (english language and male and humans and yr="2002 -Current" and english and journal article and "therapy (best balance of sensitivity and specificity)") | 1,292 |
| 4 | Concept 4: | limit 2 to (english language and male and humans and yr="2002 -Current" and english and journal article and "therapy (best balance of sensitivity and specificity)") | 11,337 |
| 5 | Search Run | #3 AND #4 | **38** |

### Database #4: PsycINFO via Ovid

| # | **Filters** | **Filters: Full-text, English, year 2002-current, NOT: review**, **book, report, single case, documents, opinion.** | **n** |
| --- | --- | --- | --- |
| 1 | Concept 1: erectile dysfunction | ('sexual activit*' or 'sexual dysfunction*' or 'sexual malfunction*' or 'sexual disorder*' or 'erectile dysfunction*' or 'hypoactive sexual desire*' or 'premature ejaculation' or 'delayed ejaculation' or 'physical discomfort').mp. [mp=title, abstract, heading word, table of contents, key concepts, original title, tests & measures, mesh word] | 21,995 |
| 2 | Concept 2: diabetes mellitus | ('Type-2 Diabetes' or 'Diabetes mellitus type 2' or 'Diabetes Mellitus' or DM or Hyperglycaemia or 'adult onset diabetes' or 'metabolic disorder*').mp. [mp=title, abstract, heading word, table of contents, key concepts, original title, tests & measures, mesh word] | 22,831 |
| 3 | Concept 3: | limit 1 to (all journals and human and english language and journal article and english and male and yr="2002 -Current") | 5,955 |
| 4 | Concept 4: | limit 2 to (all journals and human and english language and journal article and english and male and yr="2002 -Current") | 9,854 |
| 5 | Search #5 | #3 AND #4 | **154** |

### Database #5: Web of Science

| # | **Filters** | **Filters: Full-text, English, year 2002-current, NOT: review**, **book, report, single case, documents, opinion.** | **n** |
| --- | --- | --- | --- |
| 1 | Concept 1: erectile dysfunction | ALL=('sexual activit*' or 'sexual dysfunction*' or 'sexual malfunction*' or 'sexual disorder*' or 'erectile dysfunction*' or 'hypoactive sexual desire*' or 'premature ejaculation' or 'delayed ejaculation' or 'physical discomfort') |  |
| 2 | Concept 2: diabetes mellitus | ALL=('Type-2 Diabetes' or 'Diabetes mellitus type 2' or 'Diabetes Mellitus' or DM or Hyperglycaemia or 'adult onset diabetes' or 'metabolic disorder*') |  |
| 3 | Concept 3: | #1 AND #2 |  |
| 4 | Concept 4: | limit 3 to (PUBLISHED 2002-01-01 to 2022-02-02, LANGUAGE: English, DOCUMENT TYPES: articles, SUBJECTS: urology nephrology, Endocrinology Metabolism, Nursing) AND **Articles** (Document Types) and **English** (Languages) and **Endocrinology Metabolism** or **Urology Nephrology** or **Nursing** (Research Areas) | 1,333 |

### Database #6: PubMed

| # | **Filters** | **Filters: Full-text, English, year 2002-current, NOT: review**, **book, report, single case, documents, opinion.** | **n** |
| --- | --- | --- | --- |
| 1 | Concept 1: erectile dysfunction | ALL=('sexual activit*' or 'sexual dysfunction*' or 'sexual malfunction*' or 'sexual disorder*' or 'erectile dysfunction*' or 'hypoactive sexual desire*' or 'premature ejaculation' or 'delayed ejaculation' or 'physical discomfort') |  |
| 2 | Concept 2: diabetes mellitus | ALL=('Type-2 Diabetes' or 'Diabetes mellitus type 2' or 'Diabetes Mellitus' or DM or Hyperglycaemia or 'adult onset diabetes' or 'metabolic disorder*') |  |
| 3 | Concept 3: | #1 AND #2 |  |
| 4 | Concept 4: | *Filters applied:*Full text, Clinical Study, Clinical Trial, Comparative Study, Multicenter Study, Observational Study, Randomized Controlled Trial, Humans, English, Male, Adult: 19+ years, from 2002/1/1 - 2022/2/2*.* | 75 |

### Database #7: ProQuest

| # | **Filters** | **Filters: Full-text, English, year 2002-current, NOT: review**, **book, report, single case, documents, opinion.** | **n** |
| --- | --- | --- | --- |
| 1 | Concept 1: erectile dysfunction | ('sexual activit*' or 'sexual dysfunction*' or 'sexual malfunction*' or 'sexual disorder*' or 'erectile dysfunction*' or 'hypoactive sexual desire*' or 'premature ejaculation' or 'delayed ejaculation' or 'physical discomfort').  Date: From January 2002 to 2022, Source type Scholarly Journals, Document type Article, Language English | 406,447 |
| 2 | Concept 2: diabetes mellitus | ('Type-2 Diabetes' or 'Diabetes mellitus type 2' or 'Diabetes Mellitus' or DM or Hyperglycaemia or 'adult onset diabetes' or 'metabolic disorder*').  Date: From January 2002 to 2022, Source type Scholarly Journals, Document type Article, Language English | 221,743 |
| 3 | Concept 3: | limit 1 & 2 to (Peer-reviewed, Full-text, Type: Article Subject: Male, Humans, Language: English) |  |
| 4 | Concept 4: | limit 2 to (all journals and human and english language and journal article and english and male and yr="2002 -Current") |  |
| 5 | Search #5 | #3 AND #4 | **308** |
